# PERCEIVED SOCIO-EMOTIONAL BENEFITS TO GAMEPLAY FOR MILD COGNITIVE IMPAIRMENT–CARE PARTNER DYADS

**DOI:** 10.1093/geroni/igad104.3110

**Published:** 2023-12-21

**Authors:** Kara Mumma, Patricia Griffiths, Frances Harris, Maribeth Gandy, Chantal Kerssens

**Affiliations:** Georgia Institute of Technology, Atlanta, Georgia, United States; Georgia Institute of Technology, Atlanta, Georgia, United States; Georgia Institute of Technology, Atlanta, Georgia, United States; Georgia Institute of Technology, Atlanta, Georgia, United States; care.coach, Millbrae, California, United States

## Abstract

Mild Cognitive Impairment (MCI) can increase social isolation by making it difficult to engage in once enjoyable activities with others, which in turn, can exacerbate cognitive decline. Games offer stimulating cognitive and social activity, but many are not designed for individuals with MCI. A structured interview was conducted to aid in the co-design of an adaptive game for individuals with MCI and their care partners. Preliminary results on the specific barriers and facilitators affecting game play to inform design and improve accessibility were previously reported (Kerssens et al., 2020). Here we report on the perceived socio-emotional benefits identified by seven dyads who were interviewed regarding game play experience and perceptions. The dyads comprised of an individual with a clinical diagnosis of MCI (85.7% male; age range 62-85 years) and a care partner (85.7% female; age range 41-76 years). The interviews were transcribed and analyzed thematically, independently coded, and reviewed until consensus was reached. The dyads iterated many “social,” “emotional,” and “impacts” of gameplay, prompting an examination of subjective elements of game play. For example, they enjoy the simplicity and familiarity of playing games – either as a playful diversion from their everyday routine or as part of a routine. Quantitative results are reported across dyads and themes. Descriptive quotes and individual qualia are presented to illustrate specific examples of benefits and detriments to gameplay. Results are discussed in terms of the implications of game play for fostering meaningful, potentially therapeutic social activities for individuals with MCI and their care partners.

